# Temporal and spatial arrangement of wheat sowing date: a revolutionary strategy to accomplish Tianfu Granary

**DOI:** 10.3389/fpls.2023.1240417

**Published:** 2023-11-20

**Authors:** Feiquan Tan, Yulian Hou, Xinyu Huang, Jia Jia, Huai Yang, Peigao Luo

**Affiliations:** ^1^ Key Laboratory of Plant Genetics and Breeding at Sichuan Agricultural University of Sichuan Province, Agricultural College, Sichuan Agricultural University, Chengdu, China; ^2^ Sichuan Long-Gao-Fei Agricultural Science and Technology Co., Ltd, Chengdu, China

**Keywords:** crop cultivation, rice-wheat cropping, sowing date, Tianfu Granary, wheat, yield

## Abstract

Rapidly global urbanization and economic growth in the past several decades have resulted in a sharp contraction of arable areas worldwide. However, food supply requirements are quickly increasing due to higher living standards and larger populations. Therefore, food crises are still a major threat to human society. The conflict between farmland areas and the increasing need for essential supplies is becoming acuter in China. Therefore, we suggest that a novel strategy would address the issue, in which temporal and spatial arrangement of wheat sowing dates would be highly focused.

## 1 Introduction

The Tianfu Granary strategy was firstly put forward in 2022, which is a powerful policy to boost grain production in Sichuan Basin of China in the future. Rapidly global urbanization and economic growth in the past several decades have resulted in a sharp contraction of arable areas worldwide. However, food supply requirements are quickly increasing due to higher living standards and larger populations. Therefore, food crises are still a major threat to human society. The conflict between farmland areas and the increasing need for essential supplies is becoming acuter in China because of the large population and the most rapidly increasing living standards (Chen et al., 2014). Addressing this issue is a top priority for the Chinese government.

Sichuan, as a major grain-producing region in China, has always enjoyed the reputation of being the “Heavenly Land of Plenty”. From 1953 to 1969, Sichuan grain production accounted for 10% of total grain production in China (Lei, 1983); however, this amount decreased by 47.3% to only 5.27% in 2020 (National Bureau of Statistics of China, 2020).

Tianfu Granary strategy refers to the crop production region of Sichuan Province, which mainly consists of the Chengdu Plain, Anning Valley and both southern and northeastern parts of Sichuan. This region contains more than 6.7 million hectares, accounting for approximately 5.5% of the arable area of the country (Zeng and Zhang, 2003). Rice (*Oryza sativa* L.) and wheat (*Triticum aestivum* L.) are the primary summer and winter crops of Tianfu Granary and account for approximately 42% and 7% of the total grain production in the region, respectively.

Wheat production in this region involves the rice–wheat cropping system to saves land resources, which could increase crop production while reducing environmental footprint (Chai et al., 2021); however, there are numerous idle farmlands during the wheat production season within the rice-wheat cropping system, resulting in an obvious imbalance between rice and wheat production. Given that 90% of the farmlands used for rice production could be employed to simultaneously grow rice and wheat using this cropping system, the total wheat production could be increased by 165% because the wheat yield is approximately 70% that of rice in this region. Idle farmlands during the wheat production season likely exist because the yield and production cost of wheat are two important commercial factors; however, the adaptation of cultivars to seasons could be the most important biological factor.

Historically, wheat yields have increased in the region based on decreasing their plant height, increasing photosynthetic efficiency, and enhancing resistance to biotic and abiotic stresses (Luo et al., 2013), but whether a novel method for increasing wheat yield exists is an interesting topic. This was first noted by Dr. Norman won the Nobel peace prize for his achievement in wheat yield improvement, when he visited a wheat breeding station of Sichuan Agricultural University in Qionglai, Chengdu, Sichuan Province, China, on 13 May 2000. He told us: “to extend the growth period could be the most important method for further improving the wheat unit area yield of this region in the future”. Regrettably, he was unable to witness the success achieved (Swaminathan, 2009).

## 2 Analysis

**Figure 1:**
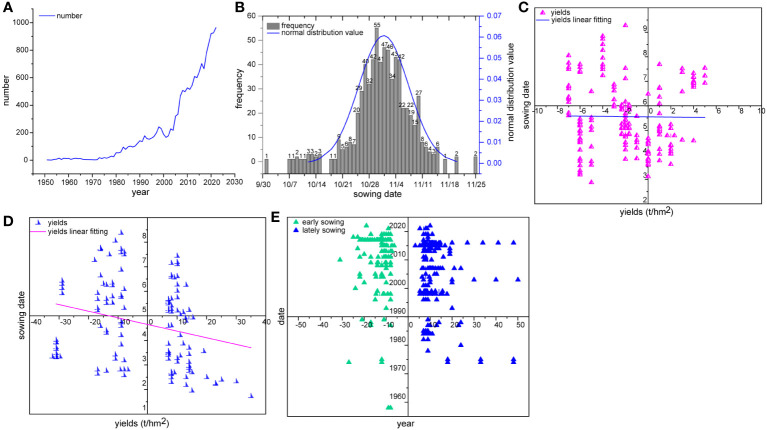
Temporal distribution of wheat sowing date and growth in the Tianfu Granary region. **(A)** The date distribution of 15,230 publications containing “Sichuan” and “wheat” in the text. **(B)** The frequency of sowing dates in normal wheat production, the data (663 times) of sowing date were drawn from 631 of 650 publications containing sowing date in the text and those (17 times) from the remaining 19 publications were not considered because they were not actually representative of normal wheat sowing data in the regions, and all sowing data were centralized around 1 Nov and 515 times from 25 Oct to 7 Nov so that we thought that the normal wheat sowing date in the region was 1 Nov, varying plus or minus 6 days. **(C)** The relationship between sowing date from 25 Oct to 7 Nov and wheat unit area yield (*r* = -0.012, *p* = 0.87, 176 data combinations from 120 publications). **(D)** The relationship between sowing date from 25 Oct to 7 Nov and wheat unit area yield (*r* = -0.252, *p* = 0.04, 124 data combinations from 80 publications). **(E)** Comparison of the frequency distribution between early and late sowing dates.

An increasing number of scientists and breeders have pay a substantial attention to changing the wheat growing season by altering the sowing date. For example, we found that 650 out of 15,230 publications from 1 Jan 1951 to 16 Sep 2022 contained “sowing date” in their texts based on searching “Sichuan” and “wheat” in the China National Knowledge Infrastructure (CNKI) and ScienceDirect databases, and 8,713 (57.2%) were published after 2010 (**Figure 1A**; **Table S1**). However, after 2010, wheat growth areas were reduced, indicating that in the past decade, many wheat scientists in the region have collectively paid their attentions on theoretically studying rather than practically studying wheat production.

In terms of production, wheat usually has a longer growth season than rice, which indicates that the adaptation of wheat to the changes of sowing date could be stronger than that of rice. We further found that the typical wheat sowing date in this region is 1 Nov with a typical range from 25 Oct to 7 Nov, and the average growth duration is approximately 190 d (**Figure 1B**). Rice is commonly sown in May, and the corresponding growth period from rice seedling planting to harvesting is approximately 130 d. Further statistical analysis of 176 combinations of sowing dates within the typical temporal range and yield data from 120 publications showed that sowing date did not have an obvious effect on yield (**Figure 1C**), while early sowing dates had a positive effect on wheat yields based on an analysis of 124 combinations of sowing dates and yield data from 80 publications (**Figure 1D**). These results suggest that there may be a substantial potential for increasing wheat yield by changing the sowing date.

Wheat is one of the most widely grown crops worldwide, while planting rice is confined to several narrow geographic regions of the eastern, southern and southeastern parts of Asia though the grown area of rice is large. Studies have shown that the latitudinal distribution of wheat ranges from 67°N to 45°S, while that of rice is only from 53°N and 34°S (Han et al., 2002; Zhao et al., 2018). Given that environmental adaptation is closely associated with the chromosome polyploid level, genome size and light adaptation (Dubcovsky and Dvorak, 2007; Amo et al., 2022), wheat could have a stronger tolerance to variation in sowing date than rice.

In fact, enhancing wheat yield by lengthening the growth period by sowing earlier has received little attention though early sowing had been suggested as an effective disease management strategy of wheat in Iran (Naseri and Sasani, 2020). Sowing on an earlier date and using a genotype that develops more slowly than others resulted in a longer growing season and consequently a significant increase in wheat unit area yield even under climate warming conditions (Van Rees et al., 2014), which could be reasonably explained with the view that sowing date of wheat had a large effect on both disease development progress and maturity duration (Naseri and Sheikholeslami, 2021; Naseri, 2022). In this cropping model, two characteristics of wheat cultivars, i.e., slow growth and no significant variation in the normal development timeframe, could be essential. In addition, in the rice–wheat cropping system of the Tianfu Granary, a year is divided into an approximately 190-d wheat growth season, a 130-day rice growth season and an average 45-day fallow interval varying from 30 to 60 days between rice harvesting and wheat sowing. Therefore, sowing wheat early and harvesting rice late would be the perfect combination for this cropping system. However, the potential of developing late-harvesting rice cultivars is very small due to the narrow adaption to developmental date, so that we focus on wheat tolerant cultivars to sowing date.

Unfortunately, in terms of early-sowing wheat cultivars, few scientists have indeed focused on sowing date except of the association of sowing date and disease epidemics (Naseri and Sabeti, 2021), but of those that have made this a focus, many would like to postpone rather than to advance wheat sowing (**Figure 1E**). The literature noted late sowing 147 times and early sowing 98 times; in addition, the longest delay in sowing date was more than 50 days, while the earliest sowing date was only 30 days early. Theoretically, under no disease stress, lengthening the growth season by sowing early would significantly increase wheat yield as wheat unit area yield is the product of unit area ear number, grain number per spike and grain weight, and these factors are predetermined before jointing, from jointing to flowering and after flowering, respectively, corresponding to 80, 60 and 50 days over the growth season, respectively. If sowing occurs 30 d early, these factors would occur 14, 9 and 7 days into the three stages. Various studies have confirmed that the unit area ear number, grain number per spike and grain weight are positively and linearly related to the biomass accumulated during their corresponding developmental process (Shamuyarira et al., 2022). Consequently, the yield potential would be increased by 55.3% (1.158^3^-1), which would surpass that of the high-yield regions in northern China. Generally, it is a common belief that northern China has a higher wheat unit area yield than southern China due to the length of the growing season. However, according to the described association of yield with sowing date, air temperature and diseases prevalence (Naseri and Sheikholeslami, 2021; Naseri, 2022), it is difficult to support this view because wheat growth has almost stagnated in the north due to low temperatures that occur for approximately 20-70 days during the corresponding season.

Moreover, the increase in yield potential amplitude could also be underestimated because there is more sunshine, precipitation, heat resources and increasing seasonal atmospheric CO_2_ in the lengthened period (Zeng et al., 2014). Early sowing practices showed that wheat cultivar Chuannong 27 sown on 18 Oct and R802 sown on 19 Oct obtained significantly higher yields than those of Chuannong 17 sown on 5 Nov and R802 sown on 2 Nov, respectively, and the corresponding amplitude increased by was 29.9% and 23.5%, respectively (Tan, 2008), thus supporting the substantial potential for improving wheat yield by sowing early.

Surprisingly, in contrast, late sowing has also garnered the attention of various scientists and farmers in the region (**Figure 1E**), and a putative explanation is that they have been concerned with cold stress in the late spring and diseases such as stripe rust (Fu et al., 2009), leaf rust (Naseri and Sasani, 2020), stem rust (Naseri and Sabeti, 2021) and powdery mildew (Naseri and Sheikoleslami, 2021). In fact, before considering these premises, we noted that in recent years, cold stress in late spring has become less frequent, causing less damage and occurring earlier in this region due to global climate warming. Meanwhile, the available disease resistance genes in the regional wheat breeding programs, such as *Yr41* to stripe rust and *Pm40* to powdery mildew, would largely reduce the risk of diseases (Luo et al., 2008; Luo et al., 2009), and even in the absence of the real resistance resources to FHB, early flowering is also advantageous to preventing pathogen infections. Moreover, the availability of both genome-edited disease resistance (Li et al., 2022) and newly transferred disease resistance gene resources such as *Fhb7* from *Thinopyrum elongatum* (Wang et al., 2020) are also conducive to improve wheat disease resistance. Thus, these concerns related to early sowing can be well addressed.

## 3 A new measure for increasing yield

**Figure 2:**
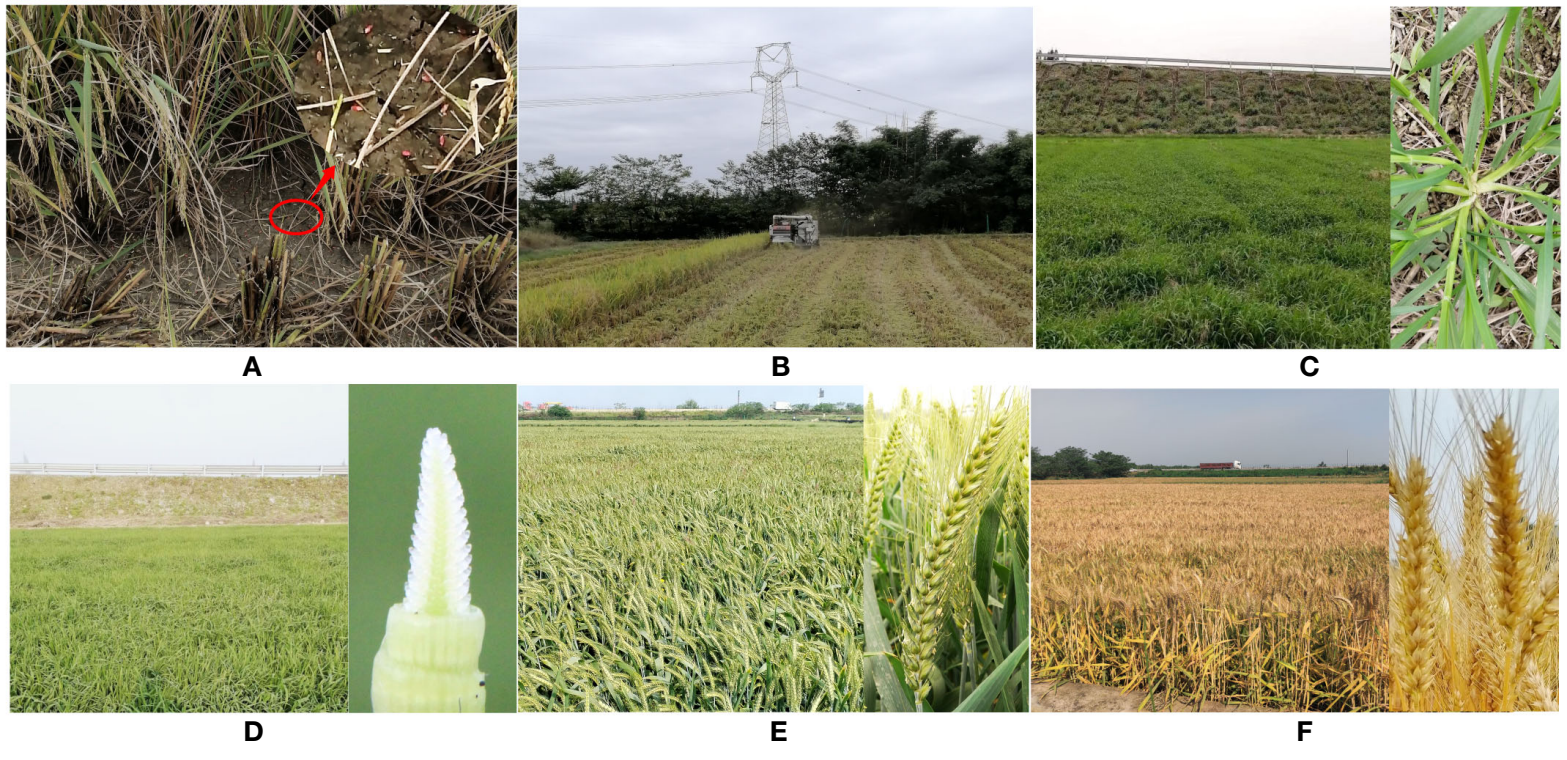
New rice–wheat intercross cropping model of sowing wheat cultivars early. **(A)** Sowing wheat seeds by unmanned aerial vehicle one or two days before the rice harvest (The red circle is zoomed in on the upper right corner of the arrow). **(B)** The automatic covering of wheat seeds by integrating the harvester and straw crusher using the reaper during rice harvesting. **(C)** In the seedling stage, the vigorous ability of wheat seedling tillers was the most important factor deterring the unit area ear number. **(D)** At the jointing stage, the large potential of seed number development was shown by the young panicle differentiation in the early wheat cultivars, the key factor influencing grain number in each ear. **(E)** During the grain filling stage, the good filling status shows the potential for each grain to have a high weight. **(F)** The mature wheat of the final high yield.

An approach for effectively using cultivars tolerant to early sowing in rice–wheat cropping systems is our last concern. By temporally arranging both dates of wheat sowing and rice harvesting, we precisely established a slight overlapping period with 2 to 5 days between them. Under these conditions, for the spatially optimal arrangement of the wheat sowing date, a new rice–wheat intercross cropping model could further be derived from the traditional rice–wheat cropping system, in which the early drainage of water was the sole essential event ensuring that the farmland successfully changes from wet to dry when rice was harvested. Following this process, we simply planted wheat seeds by unmanned aerial vehicles just one or two days before rice harvesting (**Figure 2A**), and then automatically covered them with broken straw by reapers during the following rice harvesting period (**Figure 2B**). In addition to extending the wheat growing season, there were also several advantages, such as omitting the plowing process also known as no-till, simplifying the sowing process, reducing fertilizer utilization as the soil had adequate nutrients, and reducing the use of herbicides due to effective covering by fast-growing wheat seedlings, which would be to obviously cut costs and save soil, time and fuel by integrating soil-crop system management (Chen et al., 2014). Overall, the whole growth process exhibited high yields of components, such as increased ear number (**Figure 2C**), grain number per ear (**Figure 2D**) and grain weight (**Figure 2E**), and the final wheat unit area yield was higher than that in other systems (**Figure 2F**).

## 4 Discussion

The total potential wheat production in Tianfu Granary was further estimated according to the above results and was mainly influenced by both the highest unit area yield potential and the total available farmland area in the rice–wheat cropping system for wheat growth. At present, the average and the highest unit area yields of wheat in the Tianfu Granary are 5.1 and 7.8 ton/hm^2^, respectively. Wheat unit area yield could increase by 55.3% by exploiting cultivars tolerant to early sowing so that the corresponding potential of the average and the highest unit area yields of wheat in this region would be as high as 7.9 and 12.1 ton/hm^2^, respectively. The farmland area for rice growth every year is approximately 1.9 million hectares. Given that 90% of the area is used to grow wheat as part of the rice–wheat intercross cropping model, total wheat production in Tianfu could increase to approximately 12.5 million and even possibly 20.7 million tons, which would significantly overshadow wheat production at present.

Economically, cost control is usually the final factor influencing the application prospects of a revolutionarily different system. It is evident that the effect of rice–wheat intercross cropping on cost control is very beneficial. Based on an estimation calculation, each hectare of production would save 2,130 RMB, which is the sum of 1300, 300, 450 and 180 RMB saved from the absence of plowing, simplified sowing procedure, decrease in fertilizer utilization and removal of herbicides from production, respectively. Similarly, if 90% of farmland for rice cultivation is used to grow wheat with the rice–wheat intercross cropping model, a total of 3.6 billion RMB would be saved. In addition, early sowing of wheat also decreases the carbon emission peak and shortens the time needed to achieve carbon neutrality as photosynthesis occurs for a longer duration during which carbon dioxide is effectively assimilated.

Conclusively, it is a biologically ideal choice to adjust the wheat sowing date due to its strong tolerance, and using wheat cultivars tolerant to early sowing in the rice–wheat intercross cropping system is a promising application of this system; thus, these two approaches would have a significant effect on total wheat production in Tianfu Granary, accompanied by considerable economic savings and an ecological contribution to the carbon economy. Thus, optimizing the temporal and spatial arrangement of wheat sowing dates by developing and applying wheat cultivars highly tolerant to early sowing would be a revolutionary strategy to develop for the Tianfu Granary in the future.

## Data availability statement

The original contributions presented in the study are included in the article/**Supplementary Material**. Further inquiries can be directed to the corresponding author.

## Author contributions

PL and FT conceived the idea, wrote the manuscript. YH, XH, JJ and HY collected the data and analyzed the data. YH participated in the review & editing of the manuscript. All authors contributed to the article and approved the submitted version.
